# Composite Laminate Delamination Detection Using Transient Thermal Conduction Profiles and Machine Learning Based Data Analysis

**DOI:** 10.3390/s20247227

**Published:** 2020-12-17

**Authors:** David I. Gillespie, Andrew W. Hamilton, Robert C. Atkinson, Xavier Bellekens, Craig Michie, Ivan Andonovic, Christos Tachtatzis

**Affiliations:** 1 Department of Electronic and Electrical Engineering, Royal College Building, University of Strathclyde, 204 George Street, Glasgow G1 1XW, UK; robert.atkinson@strath.ac.uk (R.C.A.); xavier.bellekens@strath.ac.uk (X.B.); c.michie@strath.ac.uk (C.M.); i.andonovic@strath.ac.uk (I.A.); christos.tachtatzis@strath.ac.uk (C.T.); 2 Collins Aerospace, Prestwick, 1 Dow Avenue, Prestwick International Aerospace Park, Ayrshire KA9 2SA, UK; 3 National Manufacturing Institute Scotland, University of Strathclyde, 85 Inchinnan Drive, Renfrewshire PA4 9LJ, UK; andrew.w.hamilton@strath.ac.uk

**Keywords:** aerospace, composite, delamination, inspection, machine learning, maintenance repair overhaul, non destructive inspection, quality assurance, re-manufacture, support vector classification, thermography

## Abstract

Delaminations within aerospace composites are of particular concern, presenting within composite laminate structures without visible surface indications. Transmission based thermography techniques using contact temperature sensors and surface mounted heat sources are able to detect reductions in thermal conductivity and in turn impact damage and large disbonds can be detected. However delaminations between Carbon Fibre Reinforced Polymer (CFRP) plies are not immediately discoverable using the technique. The use of transient thermal conduction profiles induced from zonal heating of a CFRP laminate to ascertain inter-laminate differences has been demonstrated and the paper builds on this method further by investigating the impact of inter laminate inclusions, in the form of delaminations, to the transient thermal conduction profile of multi-ply bi-axial CFRP laminates. Results demonstrate that as the distance between centre of the heat source and delamination increase, whilst maintaining the delamination within the heated area, the resultant transient thermal conduction profile is measurably different to that of a homogeneous region at the same distance. The method utilises a supervised Support Vector Classification (SVC) algorithm to detect delaminations using temperature data from either the edge of the defect or the centre during a 140 s ramped heating period to 80 ${}^{{}^{\circ}}$C. An F1 score in the classification of delaminations or no delamination at an overall accuracy of over 99% in both training and with test data separate from the training process has been achieved using data points effected by transient thermal conduction due to structural dissipation at 56.25 mm.

## 1. Introduction

The use of contact sensors and a transmission based heat source has been demonstrated to produce data which can be analysed to yield the transient thermal conduction profile indicative of inter-laminate differences between multi-ply composite laminates [1,2,3,4]. The approach provides a rapid, low energy (and thus low risk of introducing thermal damage) indication of undocumented modifications to the ply lay-up of a component yielding significant operational benefits within the Maintenance Repair and Overhaul (MRO) segment of the aviation industry. Moreover since the method is able to provide measurable differences between samples varying only in the fibre orientation between ply boundaries, it may also provide indications of other inter-laminate differences such as delaminations. Delaminations are a major cause for concern within aerospace as subsurface defects are not apparent under visual inspection [5]. Low velocity impacts can often result in delaminations beneath the surface which are much larger than the area of the impact [6,7,8]. Several established Non Destructive Inspection (NDI) techniques are able to accurately indicate sub surface defects, broadly classified as Ultrasonic [9,10,11] and Infrared (IR) Thermography [12,13,14]. Whilst these techniques are proven and reliable, the component under inspection must be removed from a repair process to either be situated within a specialist testing unit (X-ray) or treated with couplant (ultrasonic) cleaner or powders to increase emissivity (IR) [15]. Once inspected, the components then require further cleaning prior to their return to the repair process. The system developed to identify inter-laminate variance is amenable to ready incorporation within existing repair methods providing indications of delaminations that allow focused Non Destructive Testing (NDT) resulting in time savings of certified NDT inspectors and in turn financial benefit both in consumables required for NDT and staff effort. Automation of fibre orientation identification has also been achieved through transient thermal conduction profile analysis by comparison against three known fibre orientation kernel density estimate profiles and selecting the lowest mean square error [2]. The proposed method requires a single thermal profile generated from across the entire area of the sample in order to provide an indication of the inter-laminate fibre orientation changes. In order to provide useful, actionable indications of inter-laminate variances at known locations, the method must produce indications of unexpected variances to the transient thermal conduction profile for individual probes. As the sensor array is made of a grid of contact temperature sensors at known locations from both the other probes within the array and the heat source, the distances along with temperature profiles at each probe can be used as features for machine learning algorithms to identify unexpected variances against quality composite laminates.

The paper reports on the development of an approach that captures transient thermal conduction profiles of CFRP laminate panels, the basis for the accurate identification of inter-laminate delaminations. The classification of delaminations within the sample can be achieved by clustering of data—which is the grouping of data based on similarities within the groups—and the use of a suitable Machine Learning algorithm.

## 2. Experimental Set Up

**Figure 1:**
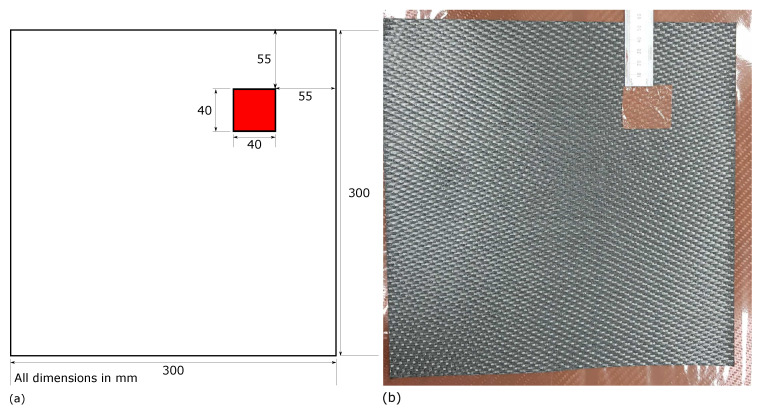
Delaminations (**a**) locations shown in red for the sample panel D8, (**b**) PTFE inclusion between the two laminate plies during lay up of sample panel D8.

A CFRP sample laminate of 300 × 300 mm in size was created from bi-axial CFRP 5 harness ply with density 1589 kg m${}^{-1}$; two plies were laid up with orientation 0/90/0/90, 0/90/0/90 to produce non-symmetrical and balanced laminate samples of 0.7366 mm in thickness.Creation of a delamination within the sample was achieved through the insertion of two Polytetrafluoroethylene (PTFE) squares 40 × 40 mm, of 0.01 mm thickness between the ply boundary of the two plies of the laminate sample at the top and right edges 55 mm from the top and right hand edges (Figure 1). The Sample was cured within Collins Aerospace’s Nitrogen pressurised Autoclave at 180 ${}^{{}^{\circ}}$C for 2 h at 35 psi. The cured samples were inspected via the tap testing method by an experienced aerospace engineer in order to ensure that a delamination had occurred. As the sample was the 8th defect variation type within a larger body of work, and in order to maintain the naming convention with the associated published data set it was assigned the label “D8” so as to allow the identification of the corresponding open data sets.

**Figure 2:**
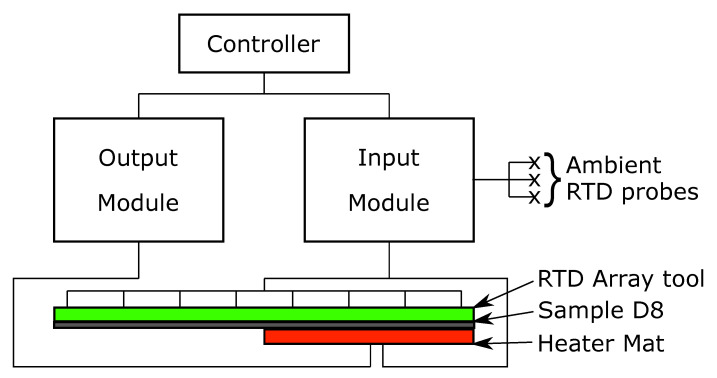
Experimental set up showing the Controller, Input module, Output module, Ambient RTD Probes, RTD array, sample D8 and heater mat.

An RTD array of RS Pro Thin Film Pt100 Platinum Resistance Temperature Detector (RTD) contact temperature sensors arranged in an 8 × 8 array with probes spaced evenly in the *x* and *y* axis by 37.5 mm, was positioned in one of two positions on sample D8, with a 150 × 150 mm heater mat positioned on the opposite surface to that of the RTD array Figure 2. The RTD temperatures were recorded by a 4-channel analogue input with the heater mat temperature powered through an output module, the temperature of which being controlled via an RTD applied to the heater mat. Ambient temperature was also recorded through 3 further RTDs attached to the analogue input module which were positioned away from the rest of the experimental set up.

**Figure 3:**
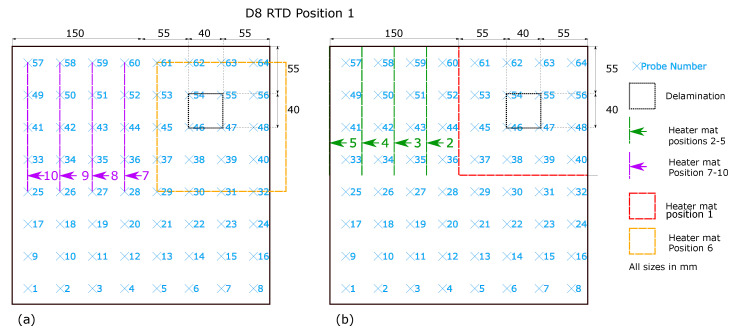
Heater mat locations for D8, displayed in dashed lines and RTD array position 1 with corresponding RTD sensors numbered with location shown in a blue ’x’ (**a**) Heater mat positions 6–10 (**b**) Heater mat positions 1–5.

RTD position 1 (Figure 3) represents the RTD array positioned centrally on the sample with an edge distance around the RTD array of 18.75 mm. The delamination is directly under 4 RTD probes, 46, 47, 54 and 55 at this RTD array position. As shown in Figure 3b a 150 × 150 mm heater mat is located in the top right quadrant of sample D8 and is applied to the opposite surface of the sample to that of the RTD array. The initial position (Heater mat position 1) matches the distance of the centre of the heater mat to the centre of the delamination. A further four heater mat positions are created for testing, shown in green, with a relocation of the heater mat in 37.5 mm increments towards the left hand edge of sample D8, yielding five unique data capture conditions. Using the same spacing between the five heater mat locations but altering the initial position by −18.75 mm in the *y*-axis and +18.75 mm in *x*-axis (Figure 3b) provides another five data capture conditions.

**Figure 4:**
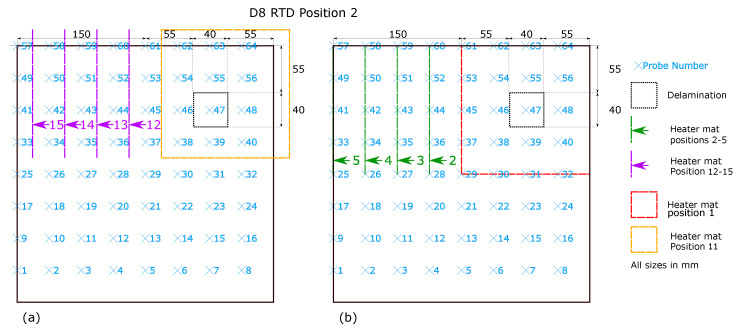
Heater mat locations for D8, displayed as dashed lines and RTD array position 2 with corresponding RTD probes numbered and locations shown in a blue ‘x’ (**a**) Heater mat positions 6–10 (**b**) Heater mat positions 1–5.

The heater mat positions 1–5 at RTD position one (Figure 3b) were replicated at RTD Position 2 shown in in Figure 4b. However the RTD array itself was relocated −18.75 mm in the *x*-axis and +18.75 mm in the *y*-axis. The relocation of the RTD array provided distances from the RTD sensors corresponding to those shown in Figure 3a heater mat positions 6–10, providing directly comparable readings. Replicating the heater mat positions 6–10 at RTD array position 2 in relation to the sample D8, produce distances from the RTD sensors corresponding to those at RTD array position 1 heater mat positions 6–10. In order to create heater mat locations in relation to sample D8 that are directly comparable to RTD array position 1 heater mat positions 1–5, a further set of heater mat positions were required, achieved by creating heater mat positions 11–15 (Figure 4a) which were offset from heater mat positions 1–5 by −18.75 mm in the *x*-axis and +18.75 mm in the *y*-axis. As shown, 4 directly comparable RTD distance from heat source positions (1–4 and 12–15) result, however positions 11 and 5 have no directly comparable heater mat positions in regards to RTDs. Thus the maximum quantity of directly comparable heater mat positions are established whilst maintaining the minimum starting edge distance of the delamination to the heat source of 37.5 mm (as seen in heater mat position 6 and 11, Figure 3a and Figure 4a respectively). The trade-off permits the maximum directly comparable RTD to heater mat positions, whilst maintaining the transient thermal conduction profiles in the *x*-axis of CFRP impacted by the delamination.

**Figure 5:**
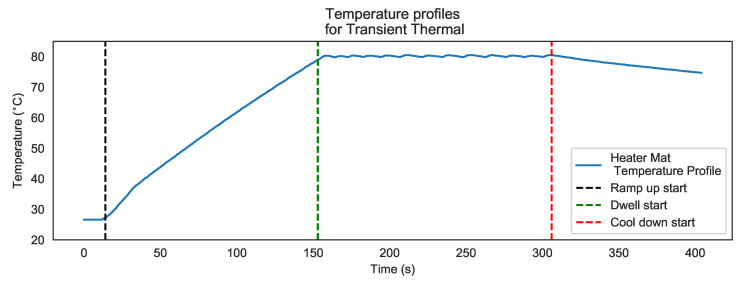
Thermal profile for heater mat and step heating profile.

Temperature against time profiles were captured and labelled according to RTD locations relative to the heater mat location and sensor positions over a delamination. In order to minimise the risk of expansion to the manufactured delamination, or the creation of new defects due to thermal cyclic loading [16,17], a low energy, low temperature (maximum 80 ${}^{{}^{\circ}}$C) heating profile was applied. The low temperature stimulus was segmented into three distinct phases: Ramp up; Dwell; and Cool down (Figure 5) over periods of 140 s, Ramp up; 160 s, Dwell; and 90 s Cool down respectively. Sample D8 was heated using the stepped heating profile at each of the heater mat locations and RTD array positions.

## 3. Initial Visualisation

The data points where plotted using the lineplot function from the python seaborn library, grouped by the ‘x_heat_dist’, the heading under which the RTD probes distance from the centre of the heater mat in mm is stored, in order to highlight any immediately obvious relationship between increasing the distance between the delamination and the centre of the heat source.

**Figure 6:**
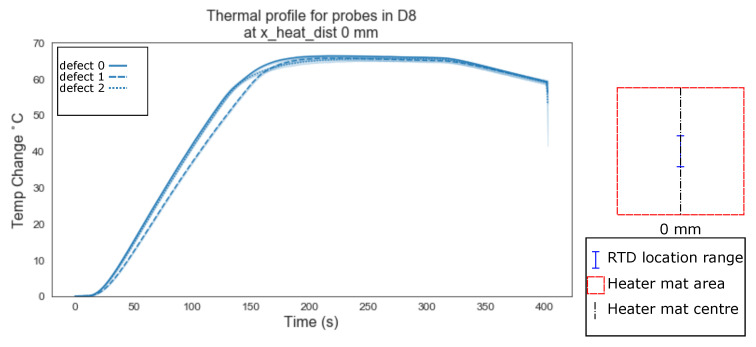
Thermal profile for probes in D8 at y_heat_dist of ≤37.5 mm and x_heat_dist of 0 mm.

**Figure 7:**
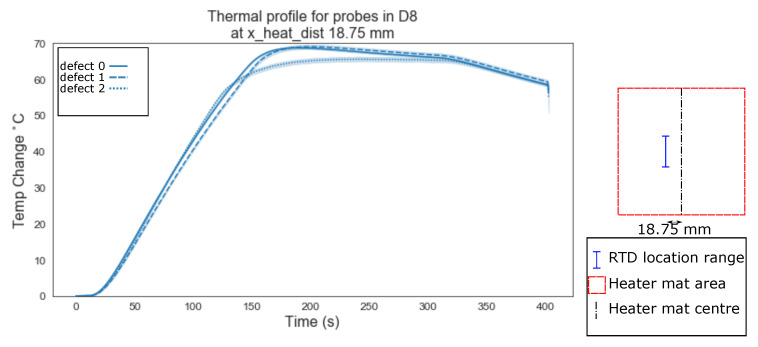
Thermal profile for probes in D8 at y_heat_dist of ≤37.5 mm and x_heat_dist of 18.75 mm.

**Figure 8:**
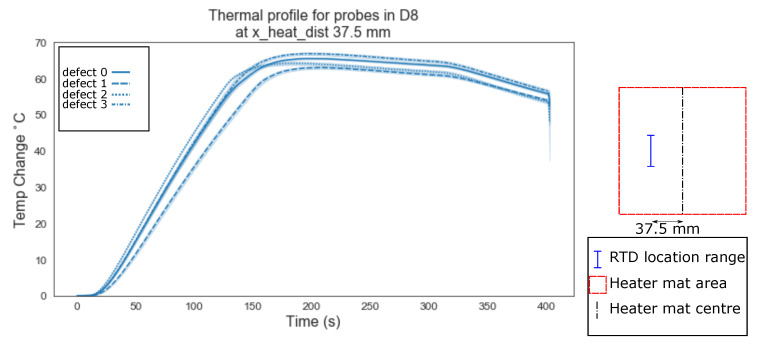
Thermal profile for probes in D8 at y_heat_dist of ≤37.5 mm and x_heat_dist of 37.5 mm.

The mean values of the temperature profiles with a confidence interval (95%) shown as the shaded area around the mean, were plotted. Figure 6 shows the variation between defects type ‘0’ (homogeneous) as a solid line, ‘1’ (edge of a delamination) as a dashed line, and ‘2’ (centre of a delaminations) as a dotted line, for RTDs located directly above the centre of the heater mat (0 mm). Figure 6, Figure 7 and Figure 8 show the mean temperature profiles for RTDs positioned directly above the heater mat varying from the centre point (Figure 6) to a quarter the heater mats width from the centre (37.5 mm, Figure 8); no immediately obvious visual similarities between defects are evident. Figure 8 shows the first instance where a defect type ‘3’ (dotted and dash mixed line) is evident. Since the delaminations is 40 mm in width, RTD distances of less than half this width mm (18.75 mm and 0 mm) from the centre of the heater mat can only be in one of two positions in relation to the delamination, positioned directly over a delamination (defect type ‘1’ or ‘2’) or have a clear path between the delamination and the centre of the heater mat (defect type ‘0’).

**Figure 9:**
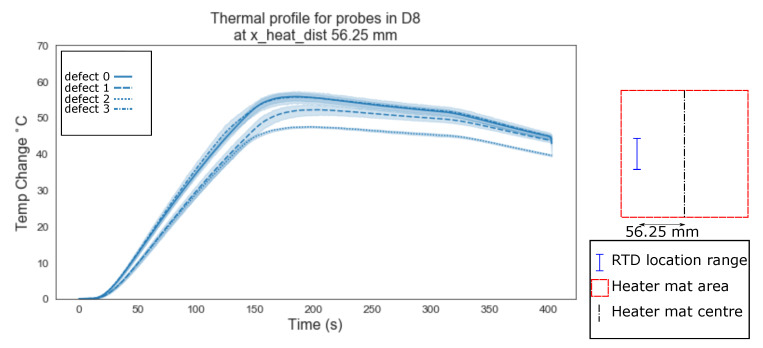
Thermal profile for probes in D8 at y_heat_dist of ≤37.5 mm and x_heat_dist of 56.25 mm.

A more consistent profile appears to emerge between the defect types ‘1’ and ‘2’ during the ramp period at the RTD distance from the centre of the heater mat of 56.25–18.75 mm from the edge of the heater mat-(Figure 9). A clearly visible reduction in the ramp rate is evident when compared to homogeneous regions ‘0’ and homogeneous regions where a defect exists in the path between the centre of the heater mat and RTD (defect type ‘3’).

**Figure 10:**
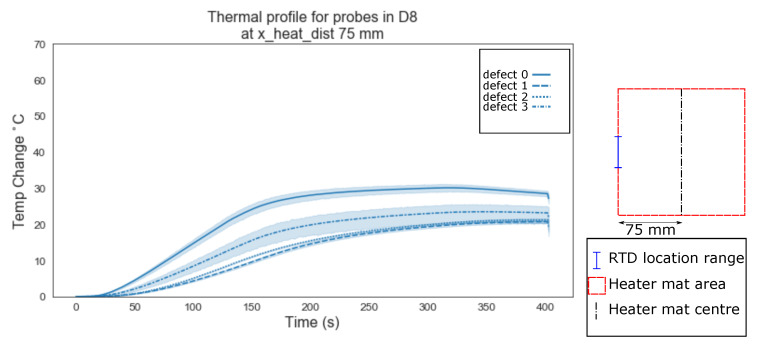
Thermal profile for probes in D8 at y_heat_dist of ≤37.5 mm and x_heat_dist of 75 mm.

The distinction in temperature profile becomes more apparent at 75 mm from the centre of the heater mat (Figure 10), at which point the RTDs are located on the boundary area of the heater mat and the unheated areas. The interaction between the heated area and unheated area is at its most obvious due to the structural dissipation of the thermal energy origination from the heater mat being drawn into the unheated sample at this sample evaluation configuration. A distinct mean temperature profile is evident when compared to the homogeneous regions (defect ‘0’), homogeneous regions impacted by a delamination in the transmission path to the centre of the heater mat (defect ‘3’) and the presence of delaminations (defects ‘1’ and ‘2’).

**Figure 11:**
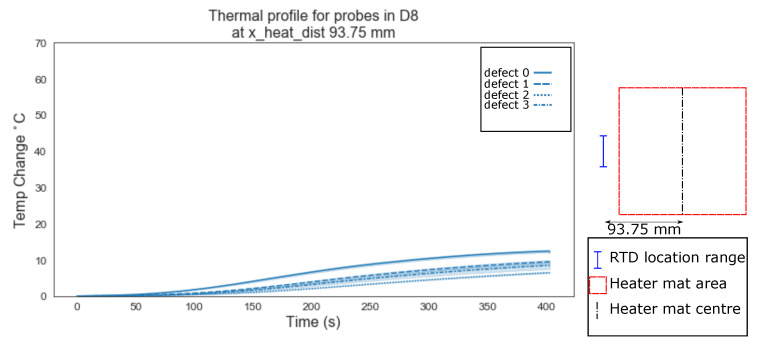
Thermal profile for probes in D8 at y_heat_dist of ≤37.5 mm and x_heat_dist of 93.75 mm.

When the distance reaches 93.75 mm from the centre of the heater mat, the temperatures measured by the RTDs are generated solely from transient thermal conduction due to structural dissipation from the heated area of the sample. The resultant mean profiles produced for the homogeneous (defect ‘0’) regions are distinct from the three defect types ‘1’, ‘2’ and ‘3’ where the mean profiles begin to overlap at the 95% confidence interval (Figure 11). The characteristic does still suggest that the transient thermal conduction will produce a measurably higher temperature against time profile when no delamination exists either at the location of the RTD sensor or in the 93.75 mm between the probe and the centre of the heater mat.

An additional increment of 18.75 mm in the distance from the centre of the heater mat—112.5 mm—also results in temperature profiles generated by transient thermal conduction attributed to structural dissipation alone. However the profiles of each of the defect types (‘0’, ‘1’, ‘2’ and ‘3’) overlap within the 95% confidence interval. The temperature profiles return to a state of no obvious visual distinction between either homogeneous or delamination.

**Figure 12:**
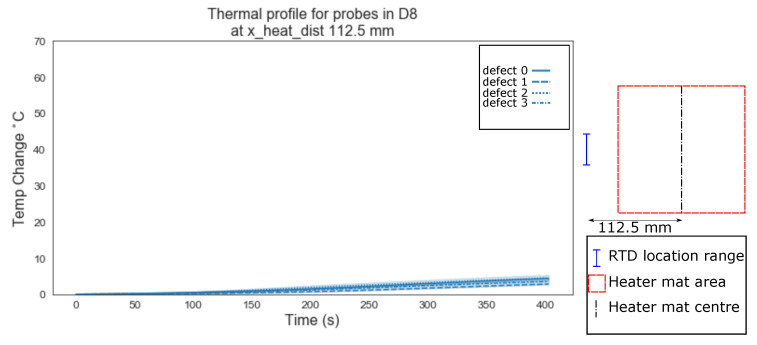
Thermal profiles for probes in D8 at y_heat_dist of ≤37.5 mm and x_heat_dist of 112.5 mm.

Evident from Figure 6, Figure 7, Figure 8, Figure 9, Figure 10, Figure 11 and Figure 12 is that the thermal profiles produced by probes situated centrally to the heater mat (37.5 mm and under) display less obvious variation in both ramp rate and maximum temperature regardless of defect type. Although the impact on the transient thermal conduction due to structural dissipation can be seen (56.25 to 93.75 mm), a reduction to the overall ramp rate and maximum temperature reached is evident only in the instance of delaminations directly below the RTD probe or to a lesser extent when the delamination exists between the probe and centre of the heater mat. In the case of the three RTD *x*-axis distances which produce visual distinctions between homogeneous and delaminations temperature profiles, these distinctions are most apparent within the 140 s ramp section of the heating profile (20 s–160 s); the resultant temperature profiles for each of these three RTD distances appear to be straight lines.

## 4. Data Acquisition and Feature Selection

For heater mat positions displayed in Figure 3 and Figure 4, sample D8 was subjected to 10 Ramp, Dwell and Cool down for RTD positions 1 and 2 repeated 10 times for each unique heater mat/RTD position combination, resulting in 12,800 temperature against time profiles. The resultant temperature against time profiles were plotted together to surface obvious outliers. The first stage in collating useful data was to remove any temperature profiles which displayed a reduction in temperature during the ramp and dwell stages, an indication of a faulty RTD sensor. The profiles were then examined visually in batches of 100 to identify profiles displaying excessive levels of noise that would be suggest that an RTD had failed or a connection issue between the probe and recording equipment. The remaining profiles had three annotations assigned to each: ‘x_heat_dist’, the distance that the probe is currently from the centre of the heat mat in the *x*-axis; ‘y_heat_dist’, the distance that the probe is currently from the centre of the heat mat in the *y*-axis; ‘defect’, if the probe is currently placed above a homogeneous region ‘0’, the edge of a delamination ‘1’, at the centre of a delaminations ‘2’, or finally if the probe is above a homogeneous region where there is however a defect between the probe and the centre of the heater mat ‘3’. The profiles where segmented further into single points of ‘Temp Change ${}^{{}^{\circ}}$C’ against ‘Time (s)’ yielding an appropriately cleansed data set of 3,833,960 rows. Three further features where added to these single points; ambient temperature readings in ${}^{{}^{\circ}}$C taken at the same instance as the corresponding data entry (‘amb_1’, ‘amb_2’ and ‘amb_3’). The use of an RTD to control the temperature within the heater mat through a feedback loop also provides another feature in the form of the ‘heater mat temperature’ in ${}^{{}^{\circ}}$C at the corresponding data entry time interval for each sample run (‘heat_mat_temp’). Given that all temperature readings are known for each sample at every time instance allows for the annotation of two additional features; the mean and median values, as these can be easily calculated for each time instance (‘mean’ and ‘median’ respectively).

The impact of a delamination on the transient thermal conduction recorded by an RTD through temperature profiles is only captured by RTDs which are inline with the defect on the *x*-axis of the technique. The instance of RTD position 1 (Figure 3) relates to RTDs 41–56, and for RTD position 2 (Figure 4) RTDs 41–48.As the most apparent differences between defect types occurs within the 20–160 s time period, the data set can be reduced to only consider values from this period. Inspection of the captured temperature against time profile for each RTD probe within the 20–160 s time period also allows calculation for the standard deviation, the final feature to the data set (‘std_140’). However restricting the data set within this range reduces the overall data set for training and validation of the machine learning algorithm to 259,299. Table 1 displays the initial five rows of the data set under the feature headings.

## 5. Machine Learning for Delamination Detection

Utilising machine learning algorithms to identify anomalies is a well established approach to categorising departures from known features. The data set created with known features is shown in Table 1 with a ‘defect’ column which has been added, depending on the presence of a delamination and it’s relationship to the centre of the heater mat, can range from 0–3. The classification of a defect is useful in order to group defect types facilitating the training and verification of the performance of a Machine Learning (ML) algorithm. However within the operational scope of an inspection process within aerospace, the inspection method is only required to indicate the presence of a defect or highlight an area of interest [18,19] and as such only two states are currently deemed of value;

- State 1: No Delamination
- State 2: Delamination

As ‘defect’ type ‘1’ and ‘2’ within the data set contain delaminations, both can appropriately be classified as ‘State 2: Delamination’; ‘defect’ type ‘0’ characterises an area that is homogeneous and as such, can appropriately be classified as ‘State 1: No Delamination’; in the case of the ‘defect’ ‘3’ class, even though type ‘3’ may be impacted by the presence of a delamination in the transient thermal conduction between the probe and the centre of the heater mat, the area itself is still homogeneous and should be described as being in ‘State 1: No Delamination’. Classification of binary set of conditions is well suited to a Support Vector Machine (SVM) algorithm implementation. An SVM algorithm seeks a hyper-plane to separate the data points within an *n* dimensional space, where *n* is equal to the number of features used within the data set [20,21,22]. The hyper-plane acts as a decision boundary, classifying points into two groups depending on which side of the decision boundary they are located. Although SVM algorithms are well suited to these classification problems, they are susceptible to reduced performance with noisy data [23], such as that which can be produced from experimentally captured data subject to external influences. In order to treat the impact of noise, SVM algorithms have a tolerance ‘C’ which produces a ‘soft margin’ to the decision boundary, allowing for the mis-classification of points. However, a balance between the number of allowable mis-classifications and defining the optimum performing decision boundary can be realised through the modification of ‘C’ during the training of the SVM algorithm. Increasing the ‘C’ value allows a higher number of mis-classifications, whilst decreasing ‘C’ reduces the number of allowable mis-classifications. Although SVM algorithms produce *n* dimensional hyper-planes, these are still in essence linear.

Furthermore, the Support Vector Clustering (SVC) algorithm [24] utilising the hyper-plane approach, applies a kernel to identify clusters of data points for classification. SVC also uses a soft margin ‘C’, but unlike the linear SVM algorithm, SVC can select a kernel which allows for new features to be created through transformations performed on the initial features of the data set. The result is the creation of non-linear decision boundaries within the *n* dimensional space, and thus more amenable to noisy data. The Radial Basis Function (RBF) kernel generates new features based on the distance of all data points against a selected data point. The impact of the generated features on the creation of the decision boundary can be controlled by the variable ‘γ’. A higher ‘γ’ value results in a high impact on the decision boundary profile; a lower ‘γ’ value results in low impact to the decision boundary profile.

## 6. Machine Learning for Delamination Detection Utilising SVC Algorithm and RBF Kernel

The development of the algorithm utilised sklearn [25], a well established ML library within the Python programming environment, which features the SVC algorithm along with the RBF kernel and means to control both the ‘C’ and ‘γ’ variables. The algorithm was trained with the ‘C’ value and ‘γ’ set to 0.1 as initial variables. In order to create a binary classification the Dataframe has an additional column added, which is generated from the content of the ‘defect’ column. The new column, ‘State’, either contains ‘Delamination’ in the instance of a ‘defect’ type ‘1’ or ‘2’, or ‘No Delamination’ in the instance of a ‘defect’ type of ‘0’ or ‘3’. The resultant Pandas Dataframe was segmented into three separate Dataframes in an 80:10:10 split creating a training data set (80%), a validation data set (10%) and a third test data set (10%) for use in the SVC algorithm. The training data set has the features shown in Table 2.

**Figure 13:**
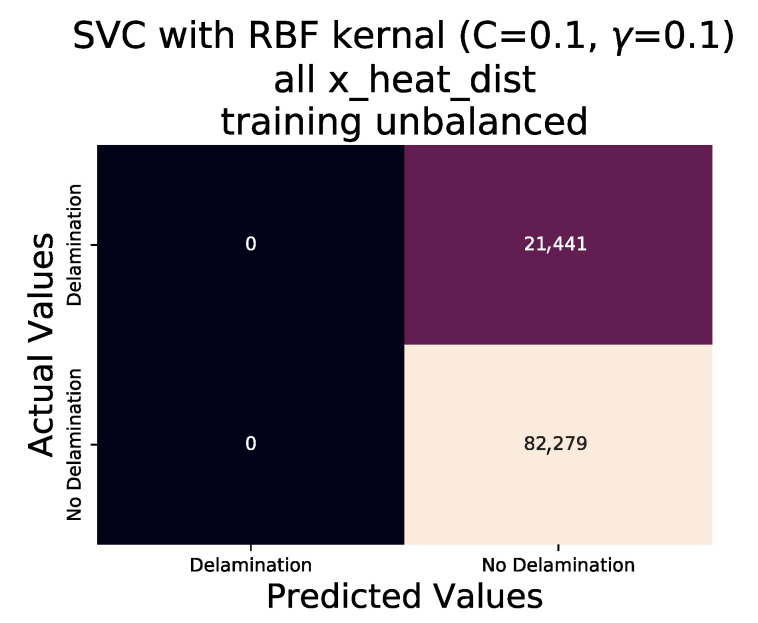
Confusion matrix of validation data set for SVC algorithm with RBF kernel, C = 0.1 and γ = 0.1, using all data points.

The corresponding classification for the data set is stored seperately in the generated column ‘State’. With ‘γ’ and ‘C’ both set to 0.1 the performance of the SVC algorithm, using the RBF kernel, when trained on all data points of the experimental training data and tested on the validation data is shown in the Classification Report (Table 3) and Confusion Matrix (Figure 13).

It is immediately clear that the algorithm performs poorly in this instance, failing completely to classify any ‘Delaminations’. The confusion matrix shown in Figure 13 demonstrates visually that the algorithm is most likely to classify a data point as ‘No Delamination’. Also evident is the existence of an imbalance between data points of classification type ‘Delamination’ and ‘No Delamination’. In total there are 21,441 ‘Delamination’ data points and 82,279 ‘No Delamination’ data points; such an imbalance in a data set often results in poor SVM performance [26]. It can therefore be argued that the poor performance also carries over to algorithms based upon SVM such as SVC. Balancing of the data set is required to test this hypothesis. The balancing is achieved by first creating two separate master data sets, the first containing only data points with the ‘State’ of ‘Delamination’ and the second with a ‘State’ of ‘No Delamination’. As the data points of ‘No Delamination’ significantly outnumber the ‘No Delamination’ data set, two options exists to achieve balanced data sets; either the ‘Delamination’ data set is artificially increased to match that of the ‘No Delamination’ or the ‘No Delamination’ has data points removed until a match to the size of the ‘Delamination’ data set is achieved. However in the goal to maintain the authenticity of the experimentally captured data, a reduction of the ‘No Delamination’ Data set was conducted. Simply reducing the ‘No Delamination’ data set to match the ‘Delamination’ data set would remove a large portion of data points with no regards as to its nature. The requirement is a reduction in the data set that also maintains comparable data, and in this instance the ‘x_heat_dist’ and ‘y_heat_dist’ features are directly comparable. Thus 3 further data sets were created from each of the two new data set (‘Delamination’ and ‘No Delamination’) which in turn contain only data points from each of the unique ‘y_heat_dist’. Repeating the process on the new ‘y_heat_dist’ separated data sets with the unique ‘x_heat_dist’ results in 7 more separate data sets for those data set featuring ‘State’ of ’No Delaminations’, 21 in total, and 5 data sets per unique ‘x_heat_dist’ for data sets featuring ‘State’ of ‘Delamination’ producing 15 in total. The 21 ‘No Delamination’ data sets were then reduced to 15 of the data sets with corresponding ‘x_heat_dist’ and ‘y_heat_dist’ values followed by reducing the number of data points within the 15 ‘No Delamination’ data sets to that of the ‘Delamination’ data set with shared values for both ‘x_heat_dist’ and ‘y_heat_dist’. Upon completion of this process the remaining ‘Delamination’ and ‘No Delamination’ data sets are concatenated into a single balanced data set of 107,442 data points.

The balanced data set was subject to the same procedure as the original data set in regards to training and testing an SVC algorithm using and RBF kernel with values of 0.1 for both ‘C’ and ‘γ’ variables in the sklearn python library. 80:10:10 split (training:validation:test) of the balanced data set was used to develop a SVC algorithm, the performance of which is presented in the classification report of (Table 4). A significant increase in the F1-score, which is a harmonic mean of Precision and Recall calculated by:

(1) $$F1=\frac{2TP}{2TP+FP+FN}$$

where TP is True Positive (correct prediction of ‘Delamination’), TN is True Negative (Correct prediction of ‘No Delamination’) and *P* and *N* are the total Positive (‘Delamination’) and Negative (‘No Delamination’) classifications within the data set respectively. This increase in classifying delaminations improves from 0 in the unbalanced data set to 0.778 in the balanced data set results.

**Figure 14:**
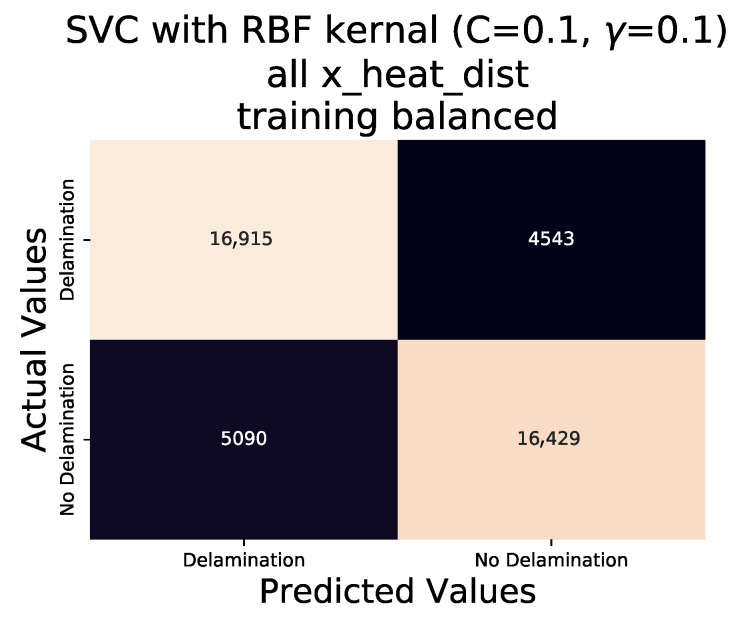
Confusion matrix of validation data set, for SVC algorithm with RBF kernel, C = 0.1 and γ = 0.1, using all data points from the balanced data set.

Figure 14 displays the confusion matrix of the SVC algorithm with the balanced data set test values. An immediate and marked improvement in the accuracy of classifications results when compared to the unbalanced data set test confusion matrix (Figure 13). Further improvements to the algorithm can be made through fine tuning of the ‘C’ and ‘γ’ values. Inspection of the F1-score, for both ‘Delamination’ and ‘No Delamination’ have increased when compared to the unbalanced data set trained algorithm; however the overall accuracy has reduced from 0.79 to 0.77. As the overall accuracy is calculated;

(2) $$Accuracy=\frac{TP+TN}{P+N}$$

A single well performing classification prediction can result in a high overall accuracy within an unbalanced data set. It is important to note that although the overall accuracy produced from a supervised trained algorithm may be acceptable, it does not necessarily provide a true reflection of the performance.

**Figure 15:**
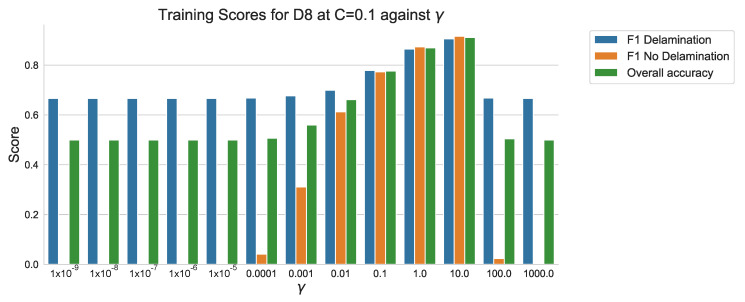
F1 scores and overall accuracy of sample D8 within training data set using SVM algorithms with RBF kernel, C = 0.1 values against γ.

The overall accuracy and F1-scores can be plotted to identify the ‘γ’ value that results in the best performance through training the SVC algorithm with incremental increases in ‘γ’. Figure 15 displays the F1 scores for ‘Delamination’ and ‘No Delamination’ as well as overall accuracy of the test samples within the training data set of sample D8. Inspection of the bar plot indicates that the highest performance is achieved for the ‘γ’ value 10.0 at ‘C’ = 0.1. Table 5 presents the resulting F1 score of 0.905 (90.5%) for ‘Delamination’, F1 score of 0.915 (91.5%) and an overall accuracy of 0.91 (91%). Although an accuracy of 100% is ideal, it is also a strong inference of algorithm over-fitting to the training data set [27,28].

**Figure 16:**
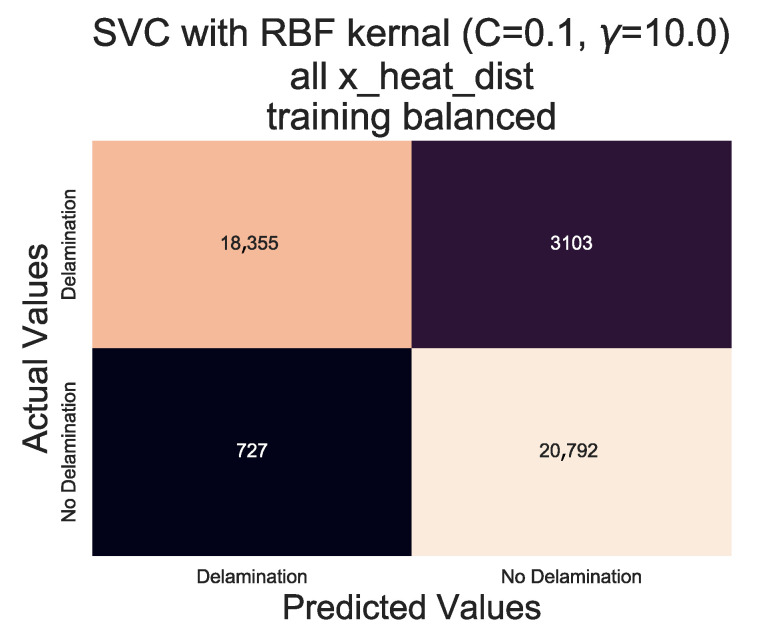
Confusion matrix for SVC algorithm with RBF kernel, C = 0.1 and γ = 10.0, using all data points from the balanced data set.

When compared to the results in Table 4, the performance of the algorithm when trained using ‘C’ value of 0.1 and a ‘γ’ of 10.0, the resultant F1 scores for both ‘Delamination’ and ‘No Delamination’ classifications show a marked improvement from 0.778 (77.8%) and 0.773 (77.3%) to 0.905 (90.5%) and 0.915 (91.5%) respectively. An F1 score above 0.9 (90%) is a significant improvement when compared to 0.8 (80%). However it does still result in a 1 in 10 mis-classification of areas with ‘Delamination’ as ‘No Delamination’, evident in Figure 16 showing that over 3000 ‘Delamination’ points have been classified as ‘No Delamination’ within the set of 21,458.

**Figure 17:**
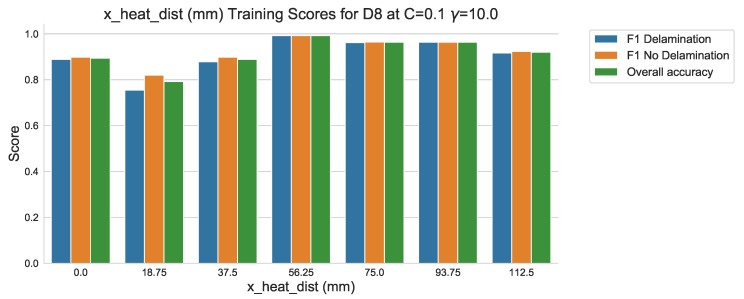
Training classification scores against ‘x_dist’.

The hypothesis regarding the performance of the algorithm trained on the balanced data set is that it is impacted by the similarities in temperature against time profiles of RTD positions 0 mm, 18.75 mm and 37.5 mm for both all types of ‘defect’ (Figure 6, Figure 7 and Figure 8). As discussed previously, a consistent difference is only apparent at the RTD positions in which the structural dissipation of transient thermal conduction appears within the temperature against time profiles of 56.25 mm and over (Figure 9, Figure 10, Figure 11 and Figure 12). The impact of structural dissipation of transient thermal conduction on the performance of the algorithm was investigated further through the testing of each unique ‘x_heat_dist’ within the algorithm trained on the balanced data set. Separation of the test values from the balanced data set by ‘x_heat_dist’ yields unique ‘x_heat_dist’ groups and the performance of each can be assessed from the classification scores recorded within each unique report. Figure 17 displays the F1 scores for ‘Delamination’ and ’No Delamination as well as the overall accuracy for the SVC algorithm when scored with the test data set from each unique ‘x_heat_dist’.

Figure 17 displays clearly that the performance of the trained algorithm when classifying validation data is at its highest at an ‘x_heat_dist’ of 56.25 mm. On comparison of the performance of probe points located closest to the centre of the heater mat (0–37.5 mm) to the performance within the areas impacted by transient thermal conduction due to structural dissipation (56.25–112.5 mm), indicates that the scores are better for the latter. Moreover, examination of the results produced by the test data set at ‘x_heat_dist’ of 56.25 mm in Table 6 highlights that the classification of ‘Delamination’ and ‘No Delamination’ is over 0.99 (99%) in all of the report headings.

## 7. Algorithm Test with New Data

As previously discussed, a high accuracy from a training data set can be an indication of algorithm over-fitting. Thus a new data set completely separated from the training data is required to test further the robustness of the algorithm. The data set 80:10:10 split for training:validation:test has been maintained throughout the training and testing of the algorithm. Thus the test data set has had no impact on the training of the algorithm and does contain the required features to be classified by the algorithm. A poor performance from the test data set would suggest an over-fitting of the algorithm.

**Figure 18:**
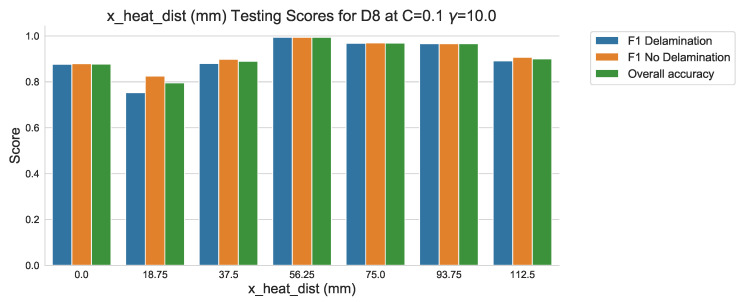
Testing classification scores against ‘x_dist’.

**Figure 19:**
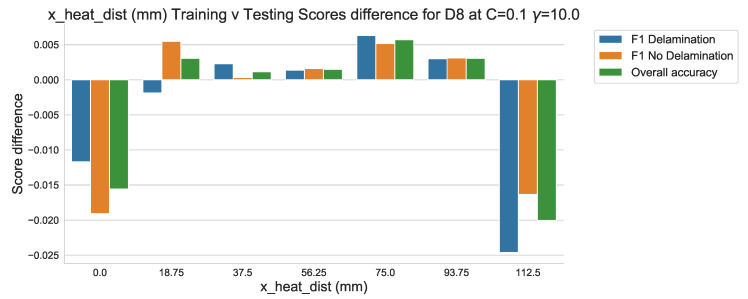
Training v Testing classification scores difference against ‘x_dist’.

The results of the classification of the testing data set in Figure 18 shows that the classification performance aligns closely with that of the validation data set used in the training of the algorithm. The difference between the two can also be quantified, as in Figure 19, by comparing the results of both the training and testing classification reports. It should be noted that although a difference in scoring does exist—expected between data sets—the margin of difference is, at it’s greatest, less than 0.025 at any score.

## 8. Application

**Figure 20:**
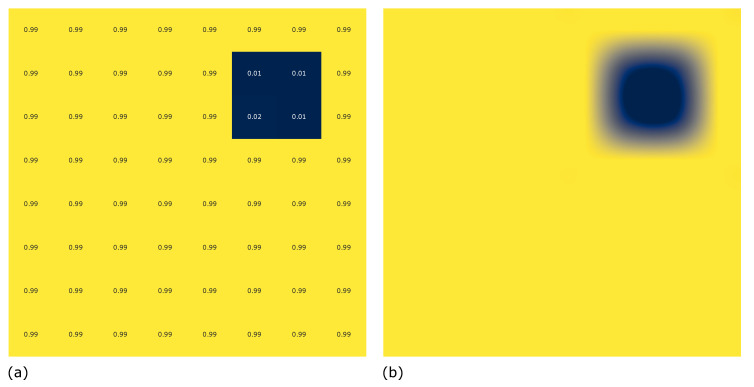
Display of overall percentage score (0–1) of the 140 time intervals for each RTD probes which classified as ‘No Delamination’ (**a**) Corresponding RTD probe locations, (**b**) Gaussian filter application to present image similar to traditional IR thermography methods.

As the proposed method has demonstrated, utilising a SVC machine learning algorithm to classify individual time intervals within the 140 s ramp period from ambient temperature to 80 ${}^{{}^{\circ}}$C produces a high level of accuracy, recall and precision. However as a standalone decision support method, the information on the degree of certainty of a delamination presented to an operator may be unclear. Presenting the captured data in a format similar to existing image based IR thermography techniques will promote a greater understanding of the underlying structure under inspection, more intuitively than in the form of confusion matrices. One approach is to present the overall percentage of scores achieved for each RTD probe at 56.25 mm distance from the centre of the heater mat in the *x*-axis over the 140 s period as seen in Figure 20a. In this instance the homogeneous regions of sample D8 produced classifications of 0.99 (99%) are clearly visible as it the area corresponding with a delamination producing scores of between 0.01 and 0.02 (1–2%).

The application of a Gaussian interpolation to the plotting of the generated 8 × 8 scoring, generates a heat map more familiar to users of established IR image based thermography techniques, as seen in Figure 20b. This presentation of the results within a report will improve the understanding of operators familiar with established techniques.

## 9. Conclusions

The paper reports on the effects on the transient thermal conduction between heated and unheated areas of CFRP laminates from the temperature against time profile produced at increasing distances from the central heating point. Heater mats applied to the sample deliver a short and low temperature step heating profile over 390 s and 80 ${}^{{}^{\circ}}$C to generate temperature against time profiles. Results show that the temperature against time profiles are effected by inter-ply delaminations at the greatest degree where transient thermal conduction occurs due to structural dissipation between the heated and unheated areas.

SVC machine learning algorithm with an RBF kernel (‘C’ = 0.1 and ‘γ’ = 10.0) was trained a using an experimentally captured data set of temperature against time profile points from an RTD sensor array, during the 140 s ramp up period and across multiple heater mat and RTD array positions. The trained algorithm produces F1 scores of over 0.99 (99%) for both ‘Delamination’ and ‘No Delamination’ classifications for the validation data sets and a further test data set isolated during the training process at distances of 56.25 mm from the centre of the heat source. As the method was developed specifically for a two ply bi-axial five harness CFRP laminate, calibration for other weave types and ply lay-ups require individual calibration.

The method utilises commonly available RTD temperature sensors and heater mats, compatible with most CFRP and other composite curing/repair processes. Operation at low temperature when compared to other more established thermography methods also reduces the risk of thermally induced damage. The proposed method and implementation provide a decision support capability for operators to rapidly identify delaminations within a CFRP laminate with readily available, low cost equipment. The information is presented in a manner which draws parallels with established IR methods so as to minimise the training requirement in order to interpret the results. In regard to the physical implementation, other commercially available sensors provide the necessary data foundation for the identification of delaminations and in turn the generation of easily interpreted NDI reports.

## Figures and Tables

**Table 1:** Pandas Dataframe of dataset showing the first five rows and column headers.

	Temp	Time (s)	x_heat_dist	y_heat_dist	std_140	Mean	Median	heat_mat_temp	amb_1	amb_2	amb_3	Defect
	Change ${}^{{}^{\circ}}$C											
0	0.1	20	75.0	0.0	6.068788	0.218182	0.1	29.8	24.5	24.3	24.2	0.0
1	0.1	21	75.0	0.0	6.068788	0.265455	0.1	30.5	24.5	24.3	24.2	0.0
2	0.2	22	75.0	0.0	6.068788	0.309091	0.1	31.0	24.5	24.3	24.2	0.0
3	0.2	23	75.0	0.0	6.068788	0.369091	0.1	31.5	24.5	24.3	24.2	0.0
4	0.2	24	75.0	0.0	6.068788	0.438182	0.1	32.1	24.5	24.3	24.2	0.0

**Table 2:** Pandas Dataframe of data set showing the first five rows and column headers of Training data set.

	Temp Change ${}^{{}^{\circ}}$C	Time (s)	x_heat_dist	y_heat_dist	std_140	Mean	Median	heat_mat_temp	amb_1	amb_2	amb_3
0	0.1	20	75.0	0.0	6.068788	0.218182	0.1	29.8	24.5	24.3	24.2
1	0.1	21	75.0	0.0	6.068788	0.265455	0.1	30.5	24.5	24.3	24.2
2	0.2	22	75.0	0.0	6.068788	0.309091	0.1	31.0	24.5	24.3	24.2
3	0.2	23	75.0	0.0	6.068788	0.369091	0.1	31.5	24.5	24.3	24.2
4	0.2	24	75.0	0.0	6.068788	0.438182	0.1	32.1	24.5	24.3	24.2

**Table 3:** Classification Report of validation data set for SVC algorithm with RBF kernel, C = 0.1 and γ = 0.1, using all data points.

	F1-Score	Precision	Recall	Support
Delamination	0.000000	0.000000	0.00000	21,441.00000
No Delamination	0.884725	0.793280	1.00000	82,279.00000
accuracy	0.793280			
macro avg	0.442363	0.396640	0.50000	103,720.00000
weighted avg	0.701835	0.629293	0.79328	103,720.00000

**Table 4:** Classification Report of validation data set, for SVC algorithm with RBF kernel, C = 0.1 and γ = 0.1, using all data points from the balanced data set.

	F1-Score	Precision	Recall	Support
Delamination	0.778363	0.768689	0.788284	21,458.000000
No Delamination	0.773293	0.783378	0.763465	21,519.000000
accuracy	0.775857			
macro avg	0.775828	0.776033	0.775874	42,977.000000
weighted avg	0.775825	0.776044	0.775857	42,977.000000

**Table 5:** Classification Report for SVC algorithm with RBF kernel, C = 0.1 and γ = 10.0, using all data points from the balanced data set.

	F1-Score	Precision	Recall	Support
Delamination	0.905525	0.961901	0.855392	21,458.000000
No Delamination	0.915665	0.870140	0.966216	21,519.000000
accuracy	0.910883			
macro avg	0.910595	0.916021	0.910804	42,977.000000
weighted avg	0.910602	0.915956	0.910883	42,977.000000

**Table 6:** Classification Report for ‘x_heat_dist’ at 56.25 mm validation data set, for SVC algorithm with RBF kernel, C = 0.1 and γ = 10.0, trained on balanced data set.

	F1-Score	Precision	Recall	Support
Delamination	0.992484	0.993978	0.990994	2665.000000
No Delamination	0.992427	0.990926	0.993932	2637.000000
accuracy	0.992456			
macro avg	0.992456	0.992452	0.992463	5302.000000
weighted avg	0.992456	0.992460	0.992456	5302.000000

## Abbreviations

The following abbreviations are used in this manuscript:

γ	Curvature of decision boundary within SVC algorithm
C	Soft margin for SVM and SVC algorithm
CFRP	Carbon Fibre Reinforced Polymer
IR	Infrared
ML	Machine Learning
MRO	Maintenance Repair and Overhaul
*N*	Negative
NDI	Non Destructive Inspection
NDT	Non Destructive Testing
*P*	Positive
PTFE	Polytetrafluoroethylene
SVC	Support Vector Clustering
SVM	Support Vector Machine
TN	True Negative
TP	True Positive
RBF	Radial Basis Function
RTD	Resistance Temperature Detector
